# Hyperbilirubinemia monitoring program in infants ≥ 35 weeks: a Brazilian quality improvement study

**DOI:** 10.1016/j.jped.2026.101502

**Published:** 2026-01-31

**Authors:** Ana Luiza Y. Grillo, Sérgio T.M. Marba, Jamil P.S. Caldas

**Affiliations:** a Programa de Pós-Graduação em Saúde da Criança e do Adolescente, Universidade Estadual de Campinas (Unicamp), Campinas, SP, Brazil; b Departamento de Pediatria, Universidade Estadual de Campinas (Unicamp), Campinas, SP, Brazil

**Keywords:** Infant, Newborn, Jaundice, Neonatal, Outcome assessment, Health care, Quality improvement

## Abstract

**Objective:**

To implement a systematic monitoring program for hyperbilirubinemia in the first week of life in newborns ≥ 35 weeks of gestational age.

**Methods:**

A quality assessment study was conducted with an estimated sample size of 385 newborns using a phototherapy utilization rate of 10 %. All newborns with a gestational age of ≥ 35 weeks admitted to a secondary general hospital well-baby nursery were included. Transcutaneous bilirubin (TcB) was systematically measured, and total serum bilirubin (TsB) was measured as needed. Patients with TcB levels above the 75th percentile according to the nomogram by Bhutani et al. (2021) were followed up after discharge from a specific outpatient clinic. The rates of outpatient follow-up clinic utilization, incidence of total bilirubin ≥ 15 mg/dL at discharge, readmission for phototherapy, and exchange transfusion were evaluated.

**Results:**

Of the 432 newborns analyzed, 53 (12.3 %) were referred for follow-up, with a return rate of 83 % (44). Twelve newborns (27.2 % of those who attended the return visit and 2.7 % of the total sample) were readmitted for phototherapy for a median duration of 36 (30–48) hours. Three infants had bilirubin values ≥ 15.0 mg/dL at discharge and did not require readmission. None of the patients required an exchange transfusion.

**Conclusions:**

The outpatient follow-up program for jaundice demonstrated high adherence, safety, and effectiveness, and reduced the rate of phototherapy use threefold.

## Introduction

Jaundice occurs in approximately 60–80 % of newborn infants, is characterized by elevated levels of bilirubin in the blood (hyperbilirubinemia), and has a benign course in most cases [1, 2, 3, 4]. However, due to bilirubin encephalopathy, current recommendations from the Brazilian Society of Pediatrics (SBP) and the two most recent guidelines of the American Academy of Pediatrics (AAP) indicate the need for all infants to undergo a risk assessment for hyperbilirubinemia at least once before hospital discharge [3,5,6].

Visual assessment of jaundice on physical examination is not reliable for estimating total bilirubin (TB) or identifying infants at risk for a rapid increase in TB, especially those with dark skin [7]. Thus, TB measurement by serum or transcutaneous methods is recommended [1,3,5,6].

Severe (TB > 25 mg/dL) and extreme (TB > 30 mg/dL) hyperbilirubinemia, if not identified and treated promptly, can cause severe permanent neurological damage, defined as bilirubin encephalopathy [5,6,8,9], whose main risk factors are the level of TB reached and the duration of exposure to bilirubin [8,9]. Therefore, accurate assessment of newborns at an increased risk of developing severe hyperbilirubinemia is now a major challenge in the prevention of acute and chronic bilirubin encephalopathy [4, 5, 6,10].

In Brazil, programs for monitoring neonatal jaundice in the first week of life have not been systematically evaluated [11]. In the last 25 years, a Brazilian university tertiary hospital has implemented a follow-up clinic to monitor neonatal jaundice during the first week of life, aiming to monitor newborn infants prone to developing concerning levels of hyperbilirubinemia. Consistent with American studies, this program demonstrated the effectiveness of implementing a well-established bilirubin screening protocol before hospital discharge, resulting in fewer readmissions for phototherapy, lower bed occupancy rates, reduced hospitalization costs, and, most importantly, improved mother-infant bonding and breastfeeding [12,13, 14, 15, 16].

Thus, this study evaluated the implementation of a similar follow-up protocol for newborns with jaundice, born at a gestational age > 35 weeks and weighing > 2000 g during the first week of life, at risk of hyperbilirubinemia, and born in a non-teaching secondary-level general hospital.

## Methods

### Type of study

Quality improvement study.

### Sample size

The sample size was assessed using a formula for estimating proportions in descriptive studies with categorical qualitative variables, setting the alpha significance level or type I error at 5 % (alpha = 0.05) (or 95 % confidence interval) and the sampling error/precision at 3 % (*d* = 0.03). Therefore, a minimum sample size was determined to be 385 newborns, based on the hospital previous pre-intervention phototherapy rate of 10 % of live births.

### Setting - pre-intervention context

The study was conducted at Santa Casa de Misericórdia de Rio Claro, São Paulo, Brazil, a medium-sized, secondary-level, philanthropic general hospital integrated into the Brazilian Unified Health System (Sistema Único de Saúde - SUS). The hospital serves as a regional referral center for a microregion in the state of São Paulo, covering the municipalities of Rio Claro, Analandia, Corumbatai, Ipeuna, Itirapina, and Santa Gertrudes, and operates under the regulation of the São Paulo State Health Regulation System. The obstetric ward has 26 beds, with a delivery rate of approximately 150 per month. The hospital has 10 beds in the neonatal intensive care unit and nine beds in the neonatal intermediate care unit.

The well-baby nursery comprises 20 beds. Three pediatricians (neonatologist or pediatric intensivist) performed daily assessments. Infants born vaginally were discharged after 24 h of life if they had no complications, whereas those born by cesarean section were discharged after 48 h. Before implementation of the study protocol, neonatal hyperbilirubinemia was monitored according to the current institutional protocol, as follows. When infants presented with visible jaundice during the first few hours of life, laboratory investigations were performed. After this initial period, only newborns between Kramer’s zones 2 and 3 were evaluated in more detail using serum TB sampling. After discharge, the newborns were referred to primary care facilities without risk stratification for hyperbilirubinemia or specific jaundice monitoring. Phototherapy was performed in a specific room in the pediatric ward, and its indications followed the recommendations of the SBP [3]. Before the intervention, phototherapy threshold TB levels were based on the SBP guideline on the management of neonatal hyperbilirubinemia in newborns > 35 weeks, considering postnatal age in hours and gestational age. In general, thresholds ranged from 8 to 17 mg/dL for 35–37 weeks and from 10 to 17 mg/dL for > 38 weeks; in the presence of specific risk factors, the threshold is reduced by 2 mg/dL.

The hospital has a small pediatric ward composed of 12 beds dedicated to the care of children aged 0 to 12 years, exclusively serving the municipality’s public health system. This structural limitation means that an increase in readmissions for phototherapy may significantly affect service capacity; therefore, reducing avoidable readmissions represents both a clinical benefit and an operational gain.

### Participant selection criteria

All infants born in the hospital were eligible, and the sample included newborns with a gestational age of ≥ 35 weeks and birth weight ≥ 2000 g who were referred to the rooming-in ward. Patients who underwent phototherapy before hospital discharge and those who returned to the outpatient clinic for clinical and laboratory evaluations of rebound jaundice after phototherapy were excluded.

Maternal and neonatal variables were used to describe the sample. Small for gestational age was defined as an infant whose birth weight was below the 10th percentile according to the INTERGROWTH-21st standards. Delayed umbilical cord clamping was defined as delayed clamping for at least 60 s after birth.

### Intervention and outpatient follow-up

Infants were evaluated with TcB measurement using the JM-103® transcutaneous bilirubinometer (Dräger Medical Inc., Telford, Pennsylvania) during their stay in the well-baby nursery and at discharge. In cases where the TcB value exceeded 13.0 mg/dL, the TB value was determined by serum measurement (TsB), obtained from a venous blood sample (two capillaries of 0.15 ml of blood each) and measured using an Olidef AG® bilirubinometer (Olidef, Ribeirao Preto, Sao Paulo, Brazil).

Those with TB ≥ 75th percentile for age in hours, according to the nomogram by Bhutani et al. [15] were followed up after discharge. Guardians signed the Free and Informed Consent Form and received verbal and written information about jaundice and the place, date, and time of the return visit. The interval between discharge and follow-up was defined based on the probability of progression described for each risk zone: newborns with TB ≥ 95th percentile returned within 24 h (or remained hospitalized for repeat TB measurement within 24 h); those between the 75th and 95th percentiles returned within 48 h; and those between the 40th and 75th percentiles were followed at a primary care unit, with return to the hospital outpatient clinic within 72 h when additional risk factors for hyperbilirubinemia were present. Newborns with TB values < 40th percentile were referred to primary care, as their risk of developing significant hyperbilirubinemia was negligible according to Bhutani et al [15]. Hyperbilirubinemia risk factors include low milk intake, blood incompatibility, positive direct Coombs test, siblings with a history of jaundice, presence of cephalohematoma and/or extensive ecchymosis, and Asian ancestry [3,5,6].

The implementation of the study protocol also changed the routine length of stay for infants delivered vaginally, extending the minimum discharge criterion from 24 h of life to 48 h of life. For cesarean deliveries, discharge was already typically performed at 48 h. This strategy aimed to ensure adequate clinical and laboratory evaluations for early stratification of the risk of significant hyperbilirubinemia and to reduce the chance of inadvertent early discharge in neonates at increased risk.

In this study, the treatment protocol for phototherapy TB thresholds after 24 h of life was defined as follows: newborns aged 35–37 [6,7] weeks with TsB ≥18 mg/dL and newborns ≥ 38 weeks with TsB ≥ 20 mg/dL. If, after 6 h of high-intensity phototherapy (≥ 30 µW/cm²/nm), TB persisted or exceeded 25 mg/dL, exchange transfusion was indicated. Infants readmitted for phototherapy were placed in a dedicated pediatric room ward, accompanied by their mothers. For hospitalized newborns, bilirubin was measured within 6 h after starting phototherapy using Bilitron Sky® Model Sky 5006 (Fanem, Guarulhos, São Paulo, Brazil). The initial measurement of TB and monitoring frequency during treatment were guided by infant age and bilirubin levels. Phototherapy was discontinued when TsB reached 10–12 mg/dL.

### Statistical analysis

Categorical variables were expressed as absolute and relative frequencies. Numerical variables were expressed as mean (standard deviation [SD]) or median (interquartile range [IQR]), according to their distribution. To assess the effectiveness of the intervention program, the following rates were evaluated, expressed as a percentage: rate of use of the follow-up clinic; incidence rate of TB ≥ 15 mg/dL at discharge; readmission rate for phototherapy; and exchange transfusion rate.

The implementation of the new protocol was preceded by theoretical and practical training of physicians and nursing teams for proper TcB and TB sampling. The final implementation stage involved online training of the hospital's teams and the city's primary care providers and emergency care units’ teams to ensure standardization of procedures and continuity of care after hospital discharge.

### Ethical aspects

The study was approved by the Research Ethics Committee (Registration No. 6697,432,250,000.5404).

## Results

**Figure 1:**
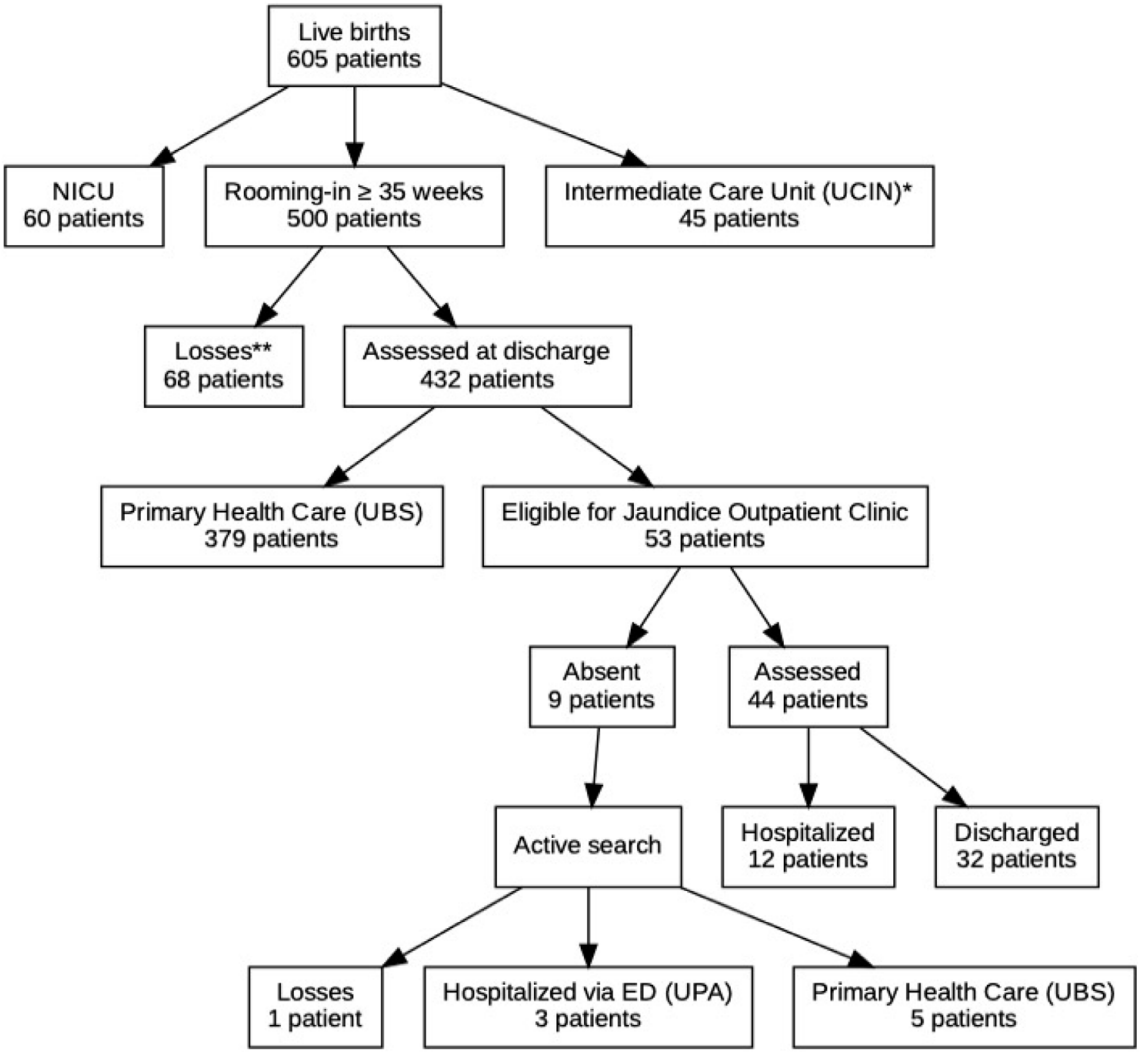
Selection flowchart of newborn infants with gestational age > 35 weeks and weight > 2000 g born between September 2023 and February 2024.

During the 5 months of the study, 605 infants were born at the hospital. Of these, 60 were referred to the neonatal intensive care unit and 45 to the intermediate care unit and were not included in the study. Of the remaining 500 patients, with a birth weight ≥ 2000 g and gestational age of ≥ 35 weeks, 432 were evaluated. The remaining 68 patients were excluded because they did not have bilirubin control at discharge, as TcB was undergoing maintenance (Figure 1).

Approximately one-quarter of mothers (22.2 %) had comorbidities, specifically diabetes mellitus (*n* = 65/15,0 %) and arterial hypertension (*n* = 51/11.8 %). Vaginal delivery occurred in 132 women (30.5 %), and delayed umbilical cord clamping was performed in 349 (80.8 %).

Most newborns were female (53.0 %), with a median (IQR) gestational age of 39 (38–39) weeks and mean birth weight [SD] of 3207 [408] g (range, 2160–4145). Among the 432 neonates evaluated, 36 (8.3 %) were classified as small for gestational age. The median length of hospital stay until discharge was 48 (42–51) hours (range, 20–168), and the median (IQR) weight loss rate at discharge was 6.5 (4.7–8.0) %.

The mean TcB level, measured in 425 newborns, was 6.8 [0.1] mg/dL (range, 0–16). The TsB values measured in eight newborns had a mean of 14.2 [0.8] mg/dl (range, 11.2–18.1).

Of the total sample, 302 newborns (69.9 %) had TB below the 40th percentile for age in hours and were referred to the primary care clinics; 106 of them (24.5 %) were between the 40th and 75th percentiles; of these, 72 (67.9 %) were also referred to the pimary care service, and 34 (32.1 %) were referred to the jaundice clinic for reassessment within 72 h because of risk factors for hyperbilirubinemia. Among these, 15 (44.2 %) had low milk intake, four (11.8 %) had blood incompatibility, eight (23.5 %) had siblings with a history of jaundice, one (2.9 %) had a positive direct Coombs test, three (8.8 %) had cephalohematoma, and three (8.8 %) were of Asian descent. Three infants (0.7 %) were in the 95th percentile, and all were referred for follow-up.

As shown in Flowchart 1, in the assessment of compliance with the study protocol, 21 infants (4.8 %) were between the 75th and 95th percentiles. Of these, five (23.8 %) were not referred to the follow-up clinic; however, an active search was conducted after discharge, and adequate follow-up at the primary care facilities was confirmed.

Of the total number of infants evaluated, 53 (12.3 %) were referred for outpatient follow-up. The return rate was 83.0 % (*n* = 44). Only one consultation was necessary for 84.1 % (*n* = 37) of the newborns, 11.3 % (*n* = 5) returned twice, and 4.54 % (*n* = 2) returned thrice. An active search was conducted for nine (17.0 %) newborns who did not return for follow-up. One of these was not found, three were admitted via the emergency care unit, and five were monitored at basic health units.

Fifteen newborns were readmitted for phototherapy (3.5 %). Their median TB value at readmission was 19.9 ± 0.1mg/dL (IQR, 17.7–20.3). Of these, 53.3 % were female, and none of the three who had TB values ≥ 15mg/dL at discharge were readmitted. Two newborns were readmitted with levels > 25mg/dL, and none > 30 mg/dL. All patients responded quickly to treatment, with a median phototherapy time of 36 (30–48) hours (range, 12–130). None of the patients required an exchange transfusion. Neurological examinations at discharge of the readmitted patients were normal. Of the newborns with bilirubinemia between the 40th and 75th percentiles at discharge, 5.6 % reached bilirubin levels ≥ 20mg/dL.

## Discussion

The implementation of structured strategies for monitoring neonatal jaundice after hospital discharge is crucial for reducing severe hyperbilirubinemia cases and preventing neurological complications. Several studies have reinforced the effectiveness of systematic outpatient follow-up programs, especially for newborns with a gestational age of 35–37 weeks, who are considered the most vulnerable [10,12,16].

Appropriate clinical guidance and risk stratification during hospitalization should be complemented by protocols that include early reassessment after hospital discharge, as advocated by Pan and Rivas [17] who highlight the importance of surveillance until 2 months of age. Home monitoring with supervised phototherapy, adopted by European institutions, has also demonstrated safety and effectiveness in preventing adverse outcomes related to neonatal jaundice [18]. Similarly, Kaplan et al. [19] highlighted the relevance of continuous outpatient surveillance systems with an emphasis on the first early return visit after discharge as a key measure for the timely detection of progressive increases in bilirubin. Such international experiences corroborate the findings of this study and highlight the importance of organizing health services to ensure the early diagnosis and timely management of neonatal hyperbilirubinemia.

This study demonstrated the feasibility of a protocol for monitoring neonatal jaundice in newborns at risk of hyperbilirubinemia, achieving a high outpatient return rate (83 %). It occurred despite challenges faced by public hospitals in scheduling discharge from joint accommodation and referral to outpatient clinics, including difficulties in transporting mothers and newborns back to the hospital. Olusanya et al. [20] highlighted the logistical challenges and need for effective strategies to ensure adequate follow-up of newborns, especially in low- and middle-income countries, where healthcare access barriers are more pronounced. Accordingly, the authors suggest taking advantage of routine immunization visits or other early postnatal opportunities to examine newborns for jaundice onset and progression.

Notably, as part of the safe hospital discharge process, besides measuring TcB and, in some cases, TsB, physicians assessed the family’s capacity to return to the hospital, and either kept the newborn hospitalized to monitor jaundice progression or, by mutual agreement, discharged the infant with scheduled outpatient follow-up [5,6,13].

Bhutani et al. [21] demonstrated that TsB and/or Tcb TB measurements before discharge can accurately predict significant hyperbilirubinemia risk, allowing early intervention and avoiding complications. Similarly, this approach is consistent with the AAP guidelines on the diagnosis, monitoring, and treatment of neonatal jaundice, which recommends assessing newborns for hyperbilirubinemia risk before hospital discharge, using TB measurement and risk factors in the previous guideline. The AAP recommends that the return interval be guided by the difference between the TB at discharge and the TB value indicative of phototherapy, including the risk factors for hyperbilirubinemia [5,6]. Additionally, the 2021 SBP guidelines on neonatal hyperbilirubinemia management recommend weighing the risks and benefits of hospital discharge when epidemiological and/or clinical-laboratory risk factors for significant jaundice are identified [3].

Guidance provided to mothers at the time of discharge is crucial to ensure adherence to the monitoring program, reinforcing the importance of follow-up and the risks associated with hyperbilirubinemia [5,13]. Additionally, rigorous monitoring is essential to prevent the development of severe hyperbilirubinemia and its complications, such as bilirubin encephalopathy [10,13,21]. Surveys conducted through questionnaires in low- and middle-income countries have shown that, although parents can recognize jaundice in newborns, they have insufficient knowledge about its causes, risk factors, and, especially, the warning signs of disease progression and complications. These include the need for hospital treatment, regardless of parental education level or parity. The authors emphasized that sociocultural motivations and traditional practices for managing jaundice may contribute to low adherence to recommended measures for the management and treatment of neonatal jaundice. [22, 23, 24] In one study, parents themselves suggested that awareness campaigns and social media programs could help raise awareness of the issue [24]. In Brazil, the Child Health Handbook–Passport to Citizenship, a book given to parents at birth, provides a brief warning about jaundice and its signs [25].

As a suggestion, neonatal units and organizations such as the SBP or nongovernmental groups that promote child health could produce brochures, apps, or posters to educate parents about neonatal jaundice. This approach has been adopted in Australia, where Queensland Health authorities produce specific educational materials for newborn parents [26]. These include informational brochures that clearly explain what neonatal jaundice is, how it presents, and the warning signs that parents should recognize. Detailed guidance is provided on phototherapy and supportive care, advising when to seek medical attention if symptoms persist. The material highlights the importance of follow-up after discharge to safeguard newborn health and ensure continuity of care.

The study also revealed a significant reduction (approximately threefold) in hospitalizations for phototherapy, supporting the effectiveness of the protocol in minimizing prolonged stays in the well-baby nursery and readmission after discharge. However, during the initial implementation phase, requests for readmission of newborns treated at Emergency Care Units increased. An online meeting was held with pediatricians from various institutions in the city, and the issue was quickly resolved. This initiative promoted greater standardization in newborn management, reinforced safe monitoring practices, and helped avoid unnecessary hospitalizations. One point highlighted by local pediatricians who participated in the meeting was that they felt more confident knowing newborns would receive adequate outpatient follow-up and that bilirubin results would be available quickly. For many, this was their first experience with TB assessment and its diagnostic and management role in neonatal jaundice. This finding corroborates the results of Bahr et al., who also observed that in a network of US hospitals, universal TcB screening before hospital discharge significantly reduced severe hyperbilirubinemia, readmissions, and the need for intensive phototherapy [27]. Furthermore, the authors noted that the protocol was well accepted by healthcare professionals, who reported greater safety and confidence when making clinical decisions based on objective data, favoring more standardized and efficient care.

This study demonstrates the feasibility of implementing a systematic jaundice monitoring and follow-up protocol in a secondary hospital, resulting in a significant reduction in hospitalizations for phototherapy and no cases of exchange transfusion, with results like those of other studies published in Brazil [12,13]. The protocol proved effective in ensuring that all newborns, regardless of socioeconomic status, had access to standardized and safe care.

In a subsequent study, it would be valuable to explore the impact of continuing education for healthcare professionals to ensure proper adherence to protocols across different settings. Integration of health systems, as observed in the study, ensures continuity of care, which is crucial to program success and enables rapid and effective interventions, avoiding complications associated with hyperbilirubinemia and improving newborn outcomes [3,6].

Another noteworthy point is the fixed phototherapy thresholds used in this study, which facilitated the health team's adherence to phototherapy indications. The higher thresholds, compared with those of the SBP, were like those currently recommended by the AAP [6]. This protocol proved safe and was associated with a reduced rate of phototherapy, as demonstrated by Balasubramanian et al. They measured the impact of the new AAP guidelines for monitoring and treating hyperbilirubinemia in newborns aged ≥ 35 weeks and found a 50 % reduction in phototherapy use and a 35 % reduction in readmissions compared with the 2004 and 2022 guidelines [28]. Digital decision-support tools based on AAP nomograms (e.g., Bilitool) are widely used in some settings; however, local practice in Brazil is primarily guided by SBP recommendations, which differ from AAP thresholds.

The extension of the minimum length of stay to 48 h may also have contributed to the observed reduction in phototherapy utilization. Because bilirubin levels physiologically rise after 24 h, a longer in-hospital observation window may have improved the identification of upward bilirubin trajectories and enabled timely risk stratification and counseling before discharge. Therefore, the reduction in phototherapy readmissions should be interpreted as the combined effect of systematic bilirubin screening, structured follow-up, and modified discharge timing.

This study has limitations that should be considered when interpreting the results. First, the absolute number of key outcome events, particularly readmissions for phototherapy, was small, which reduces the precision of effect estimates and limits more robust comparative or multivariable analyses. Second, although the return rate among those referred for follow-up was high, 17 % of referred newborns did not attend the scheduled visit. Active searching allowed the clinical trajectory of most of these infants to be identified; however, one case could not be located, and it is not possible to ensure that all absent infants evolved similarly to those who returned, which may introduce bias. Third, the initial implementation phase was subject to operational failures (e.g., non-referral of some newborns in intermediate-high risk zones), although follow-up was subsequently confirmed. Finally, this was a single-center study in a public secondary hospital, and reproducibility in other settings depends on local care flows, resources, and team training.

In conclusion, implementing a structured outpatient follow-up program for neonatal jaundice in a secondary public hospital was feasible and was associated with high adherence and no exchange transfusions, while reducing readmissions for phototherapy during the study period.

## Funding sources, or name of institutions or companies providing equipment and materials, if applicable

Not applicable.

## Data availability statement

The data that support the findings of this study are available from the corresponding author.

## Conflicts of interest

The authors declare no conflicts of interest.
